# Prof. Franco Mandelli Leukaemia Visionary. May 12, 1931– July 15, 2018

**DOI:** 10.1038/s41375-018-0352-1

**Published:** 2019-01-11

**Authors:** Sergio Amadori, William Arcese, Giuseppe Avvisati, Robert Peter Gale, Francesco Lo-Coco

**Affiliations:** 1 Vice President, GIMEMA Foundation, Rome, Italy; 2 University Tor Vergata, Rome, Italy; 3 Campus Biomedico University, Rome, Italy; 4 Imperial College London, London, UK; 5 University Tor Vergata, Rome, Italy

**Keywords:** Cancer, Medical research


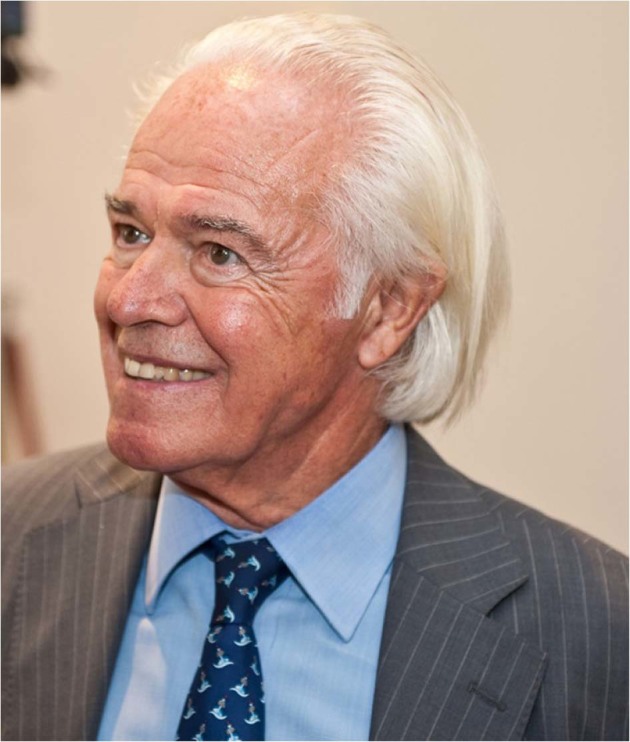


Professor Franco Mandelli passed away on July 15 in Rome, Italy, aged 87. Regarded as a father of modern Italian hematology he was a pioneer, active for decades in diverse areas of hematology and especially in the treatment of acute leukemias.

Born in the northern Italian town of Bergamo, Franco Mandelli graduated in medicine from the University of Milan in 1955 and subsequently joined the School of Internal Medicine in Parma led by Professor M. Bufano, who invited him to move to Rome in 1957. Although one of the chairs of Internal Medicine at the Policlinico Umberto I of the University Sapienza Roma had been held by G. Di Guglielmo (who first described erythroleukemia), there was at the time no school of hematology and Bufano encouraged Prof. Mandelli to start one.

After his initial interest in coagulopathies and platelet disorders, Prof. Mandelli began work on acute leukemias. Cure of these diseases was considered hopeless at that time. Encouraged by the early, albeit limited, successes of Profs. Emil Frei, James Holland, Joseph Simone and others in the US and Jean Bernard and colleagues in France, Mandelli focused on intensive therapy of acute leukemias. A critical element of his early was frequent encounters with Bernard who he often visited at Hospital Saint Louis in Paris.

During the 1970s and 1980s, Professor Mandelli organized an international conference focused on acute leukemias held in Roma at the Cavalieri Hilton overlooking the city. When he proposed the idea of the Congress some colleagues suggested the topic was too restrictive and very few hematologists would attend. They were wrong. The 1^st^ conference, held in 1973, was attended by over 400 specialists, including Europeans and Americans. Four meetings followed, the last in 1991 attended by over 1000 delegates. RPG recalls arriving for his presentation at one meeting. We used Kodak slides then and I remember rushing to the meeting room at the last moment and handing in my carousel. Franco was most forgiving of my lateness unfortunately but, unknown to me the cavernous conference hall used back projection such that every slide appeared in reverse. Image trying to explain a survival curve going left-to-right instead of right-to-left. The visual impression is dying increases survival. Probably at that early stage in my career it made little difference. Or perhaps my presentation was especially appreciated by colleagues with dyslexia or with a rear-view mirror. Forgivingly (he was always a most generous person) Prof. Mandelli ensconced me in the Presidential suite (no other rooms were available) whose size was so enormous that he and I had a brief football match after a few drinks the final conference evening.

In 1993, Prof. Mandelli also started the *International Symposium on Acute Promyelocytic Leukemia*, another Roman meeting that soon became a tradition. This was organized every 4 years until 2017. The last one was attended by more than 300 hematologists from across the world.

Among his pioneering activities, Mandelli anticipated the feasibility and potential of studies conducted by large cooperative groups. He founded the Italian multi-centre group GIMEMA in 1982 and lead it for many years. Few remember the acronym GIMEMA initially stood for *Gruppo Italia Meridionale Emopatie Maligne dell’Adulto*, because most member centres were in southern Italy. GIMEMA soon became one of the strongest multi-centre cooperative groups internationally and many institutions in the north joined. This must have pleased Dr. Mandelli coming from Bergamo and the acronym was soon changed to *Gruppo Italiano per le Malattie Ematologiche dell’Adulto*. Same acronym, different meaning. It helps to be flexible. GIMEMA’s international collaborators eventually included the EORTC, PETHEMA (Spain) and the AMLSG and SAL (Germany) cooperative groups.

Professor Mandelli had a genuine interest for research and innovation in hematology and a straightforward, forthright personality. Above all, he was a superb physician who made patients and their needs central to his activities and vision. Commemorating him on the day he died Sergio Mattarella, President of the Italian Republic, recalled Professor Mandelli: *Amongst those who contributed to the improvement of our country*. His legacy lives on through the work of the many Italian hematologists he mentored during his long and extraordinary career, including the authors below. And in the work of many international colleagues who benefitted from his wise counsel. He will be greatly missed.

